# Hydrothermal Synthesis of Kaolinite Group Minerals

**DOI:** 10.3390/ma18030472

**Published:** 2025-01-21

**Authors:** Tatiana Koroleva, Boris Pokidko, Ivan Morozov, Anastasia Nesterenko, Sofya Kortunkova, Mikhail Chernov, Dmitry Ksenofontov, Victoria Krupskaya

**Affiliations:** 1 Institute of Geology of Ore Deposits, Petrography, Mineralogy and Geochemistry, Russian Academy of Science (IGEM RAS), Staromonetny Per. 35, 119017 Moscow, Russia; pokidko2000@mail.ru (B.P.); ivan.morozov@yandex.ru (I.M.); 555stasy@bk.ru (A.N.); krupskaya@ruclay.com (V.K.); 2 Geological Faculty, M. Lomonosov Moscow State University, Leninskie Gory 1, 119991 Moscow, Russia; kortunkovasa@my.msu.ru (S.K.); miha.chernov@yandex.ru (M.C.); ksen53@gmail.com (D.K.)

**Keywords:** synthesis, precursors, kaolinite, crystallinity index, morphology

## Abstract

Synthetic alumosilicates are used in many industrial applications, and the synthesis of clay minerals under different conditions allows us to understand the conditions of their formation. This study examined the impact of varying silica precursors, pH conditions and synthesis durations. Synthetic kaolinite group mineral analogues were investigated by X-ray diffraction, scanning electron microscopy and infrared spectroscopy. Additionally, the crystallinity index was calculated. The impact of using different silica sources on the structural features of synthetic kaolinite group analogues was revealed. The use of a Nanosil precursor resulted in the formation of highly crystalline kaolinite. The most significant alterations in the course of synthesis were observed at different pH values. The formation of various synthetic analogues of minerals from the kaolinite group was observed: at a high pH, the formation of halloysite with a small admixture of kaolinite was observed. Conversely, the synthesis resulted in the formation of ordered kaolinite at a low pH. The crystallinity index of the resulting synthesized kaolinite analogues rises as the synthesis duration increases, while the quantity of non-crystallized material decreases. The changes in the crystallinity of kaolinite when using different silica precursors are related to the different homogenization of the material that occurs at the stage of alumosilica gel formation.

## 1. Introduction

Obtaining synthetic layered silicates from the kaolinite group is a fairly well-studied area of research [1]. It is known that the synthetic approach makes it possible to obtain target inorganic and hybrid materials possessing not only a given stoichiometry, but also different morphologies (plates, spherical, etc.) [2,3].

This interest in the synthesis of alumosilicate phases, synthetic analogues of natural minerals, is associated with research aimed at a better understanding of the pathways and conditions of mineral formation, as well as at the synthesis of new structures and particles with various morphologies that can find industrial applications in fields such as medicine, catalyst synthesis, etc. [1,4].

One of the most important factors determining the structure and properties of the final materials in the synthesis of inorganic particles and materials is the type of precursors (or synthons) used as initial components for the synthesis, as well as the conditions under which the maximum homogenization of the components occurs. In the case of kaolinite synthesis, it is necessary to combine two precursors (one for Al_2_O_3_ and the other for SiO_2_). The products synthesized are also affected by the temperature, pH, Si/Al ratio and synthesis duration [5,6,7,8,9].

The conventional approach to the synthesis of layered alumosilicates, including kaolinite, involves ready-to-use, finely dispersed oxide particles. After mixing, the system was treated with long-term hydrothermal conditions, i.e., using autoclave technology, since kaolinite formation is kinetically difficult under normal conditions.

One of the main ideas in many approaches is to obtain highly dispersed and homogeneous alumosilica (aluminum and silica) gels during the first stage of synthesis. Also, high dispersity should be reached to increase the solubility of the components under synthesis conditions (it might also be achieved aby increasing the synthesis temperature—200–400 °C). Homogeneity creates conditions for obtaining crystals with the required composition, preventing the segregation of the components (formation of individual oxide phases) and increasing the probability of the formation of the required phase.

In early works, commercially available silica gels (powdered amorphous silica) and prepared Al_2_O_3_ or boehmite (Al(OH)_3_) particles were used as precursors [6]. Obviously, this method initially creates a high degree of heterogeneity in the system. In order to increase their homogeneity, preliminarily obtained composite alumosilica gels could be taken as the initial material for synthesis. Ex situ, alumosilica gels obtained by the co-precipitation of sodium polysilicate with aluminum sulfate or oxychloride, followed by the removal of the electrolyte, were used in autoclave synthesis [4]. In a similar way, sodium metasilicate was used as the silica precursor and aluminum nitrate as the aluminum oxide source [9,10]. A higher degree of homogeneity can be achieved if ready-made nanoparticles are used as precursors, i.e., silicasols, in the case of silicon dioxide, and alumasols, in the case of alumino oxide. The use of silicasol (Ludox) in combination with aluminum nitrate for the synthesis of kaolinite was proposed in 1975 [11]. The simultaneous use of silicasol (Snowtex N) and aluminasol (Aluminasol-200) as precursors was proposed in [12]. Apparently, both sols contain positively charged nanoparticles (sizes up to 100 nm), which facilitates their mutual compatibility. Finally, there is the synthesis direction associated with the use of monomeric compounds from aluminum and silica as precursors, which is typical for so-called sol–gel technology. Such compounds are actively used for the synthesis of various alumosilicate materials [13]. For example, sol–gel synthesis of kaolinite was described in [14]. It involved the co-precipitation of hydrolysis products and the co-polycondensation of tetraethoxysilane (TEOS) and aluminum isopropoxide (IPA) in an aqueous media, followed by the evaporation of the solvent, alumosilica gel formation and subsequent hydrothermal treatment.

The main difficulty in producing homogeneous alumosilicate gels for subsequent autoclave synthesis of kaolinite is a significant difference in the stability and chemical activity of precursors in aqueous media at different pH values, which leads to phase separation during the early stages of synthesis. It is well known that the region of existence for aluminum in monomeric form corresponds to the region with low pH values (<3). At higher pH values, all cations are hydrolyzed to form polymeric hydroxides and oxides and will form precipitates at a neutral pH. On the contrary, in the case of silica, which only exists as an anion in aqueous media, significant solubility can only be reached at very high pH values, when it exists in the form of polysilicate anions with an average degree of polymerization of about 20–100.

However, any soluble aluminum salt can be used as an aluminum precursor in acidic conditions in aqueous sol–gel synthesis. Aluminum nitrate is the only appropriate one because nitrogen can be readily removed during subsequent heat treatments, whereas sulfate and chloride anions may remain in the final material.

In the case of silica, there is a lack of water-soluble precursors in both the acidic and neutral regions (the exception is the hydrolysate from amino-propyltriethoxysilane, APTEOS [15], which was not used for the synthesis, apparently due to its high cost). So, commercial silicasoles containing up to 50% silica are a good variant to deal with.

When ready-made, stabilized silicasol and aluminum salt are used, the mechanism of interaction includes the gradual hydrolysis and condensation of aluminum aquacomplexes on the surface of silica nanoparticles during the gradual addition of neutral or slightly alkaline sol to an acidic aluminum salt solution.

With a subsequent increase in pH, any residual aluminum in solution will precipitate to form the final alumosilica gel used in the subsequent hydrothermal synthesis.

Furthermore, the simultaneous use of silicasol and aluminasol can facilitate a high degree of homogenization. Nevertheless, phase segregation might occur during the mixing and solvent removal stages.

**Figure 1:**
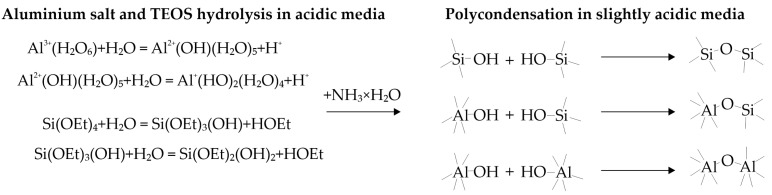
Basic reactions leading to formation of aluminosilica gel using TEOS and aluminum nitrate [17].

In the case of sol–gel kaolinite synthesis, alkoxides are rather expensive. These compounds are insoluble in water and require polar organic solvents as media. In [16], aluminum nitrate was used as an aluminum precursor and TEOS in an ethanol mixture was added dropwise to the acidic solution. The simultaneous polycondensation after ammonia addition is presented in the following scheme (Figure 1).

An alternative approach could be proposed that includes the use of so-called aminosilicates (ASs) as a water-soluble precursor to silica. This precursor can easily be obtained by dissolving silica in concentrated aqueous solutions of strong bases, such as amines [18]. The use of such a precursor makes it possible to remove nitrogen-containing components from the material by a subsequent heat treatment without using high temperatures (opposite to the case of sodium polysilicate precursors).

**Figure 2:**
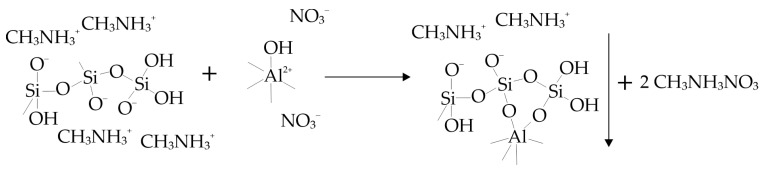
Scheme for aluminosilicate particle formation upon addition of aminosilane solution to acidic aluminum nitrate solution.

It can be expected that, when aminosilicate is used as a precursor, the addition of a concentrated and highly alkaline aminosilicate solution to an acidic aluminum nitrate solution will result in the instantaneous formation of aluminum hydroxide nanoparticles and their envelopment by a polymeric molecule (silica–oxygen polyanion) undergoing further polymerization (Figure 2).

Thus, the aim of this work is to carry out aqueous kaolinite synthesis during the first stage of kaolinite production, including sol–gel synthesis, silicasol synthesis and aminosilcate synthesis. Also, the aim is to verify optional conditions that will provide a homogeneous distribution of components. Additionally, the influence of autoclave-treatment conditions on the structure and morphology of the final synthesized particles was investigated.

## 2. Materials and Methods

### 2.1. Synthesis of Kaolinite

In the present work, three different types of silica precursors were used for kaolinite synthesis at the stage of alumosilicate gel production (the stage before autoclave treatment). In all cases, aluminum nitrate was used as the aluminum oxide precursor, from which an aqueous solution with a concentration of 5.23% wt. Al_2_O_3_ and a pH of <1 was prepared. The amounts and ratios of components were calculated based on the target composition of the final material (kaolinite) represented by the known chemical formula: Al_2_[Si_2_O_5_](OH)_4_. According to the crystallochemical formula, the molar ratio of Al/Si is 1:1 (which corresponds to the mass content of the oxides—39.5% Al_2_O_3_ and 46.5% SiO_2_).

**Figure 3:**
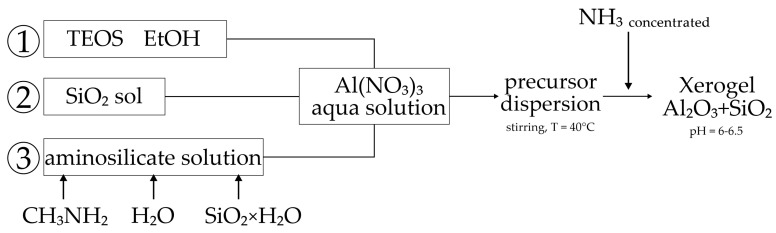
Scheme of first stage of kaolinite synthesis using different Si precursors.

1. According to the first method, the conventional sol–gel silica precursor tetraethoxysilane (TEOS) was used for the synthesis. This approach involved the preparation of an alcohol solution and then a water–ethanol emulsion of TEOS, which was then added to the aluminum nitrate solution (Figure 3(1)).
2. In the second method in the synthesis, commercial silicasol (with an average particle size of 14 nm) was used as the silica precursor. The initial silicasol, with a pH of ~9.0 and sufficient stability to acidification, was added to a concentrated aqueous solution of aluminum nitrate. Subsequently, the hydrolysis and polycondensation of aluminum was conducted in the presence of silica nanoparticles (Figure 3(2)).
3. The third method involved the use of a pre-prepared concentrated alkaline methylamine silicate solution as the silica precursor. This solution was obtained by dissolving commercially available colloidal silica in a concentrated aqueous methylamine solution. The subsequent synthesis of the aluminosilicate gel was carried out by adding an alkaline aminosilicate solution to an acidic aluminum nitrate solution, followed by co-polycondensation of the precursors (Figure 3(3)).

Commercial aluminum nitrate nonahydrate, Al(NO_3_)_3_·9H_2_O (manufacturer—the JSC Khimreaktivsnab, Ufa, Russia; synthesis grade of 98% and Mr = 375.13 g/mol), containing 13.59% Al_2_O_3_ was used as aluminum oxide precursor.

The silica precursors used were as follows:

- TEOS (Mr = 208.33 g/mol, density of 0.94 g/mL and SiO_2_ content 28.84%). The alkoxide was distilled under vacuum to remove hydrolysis products.
- Commercial colloidal silicasol-Nanosil-40M (Silicon LLC, Ekaterinburg, Russia, containing 511 g/L of SiO_2_; density of 1.303 g/mL; average particle size of 14.2 nm and pH = 9.5 according to the manufacturer’s data).
- Concentrated solution of SiO_2_ in methylamine was prepared by dissolving aqueous silica (SiO_2_·nH_2_O, LLC Trade House Reahim, Mias, Russia, LOI—26%) in 38% aqueous methylamine solution (LLC Himbaza, Moscow, Russia). The final methylaminosilicate silica concentration was 12.33%, with solution density of ~1.1 g/mL and pH > 13.

The exact proportions of the main components used in the synthesis are given in Table 1.

The silica precursor was added to the aluminum nitrate solution at a rate of about 1 mL/min while stirring and heating the solution to 40 °C (Figure 3). For the TEOS synthesis, ethanol was added to the system after the addition of the precursor to ensure homogenization (ethanol:TEOS volume ratio 4:1). In all cases, the system was homogenized by stirring on a magnetic stirrer for 60 min after the addition of the precursors. The pH values (before adding ammonia) as follows: TEOS—1.3, Nanosil—1.25 and AS—2.93.

A concentrated solution of NH_3_ (high grade, 25%) was slowly added drop by drop (at a rate of about 1 mL/min) to the solutions/dispersions after stirring for 60 min. Alkali addition was carried out under pH control until gelling occurred at pH 6–5.5. The pH was monitored by potentiometry (Ecotest-120 pH meter, calibrated with standard pH buffer solutions). The amount of ammonia solution added varied significantly depending on the type of precursor. After alkalizing the systems, gels (bulk precipitates) were formed and dried in a convection oven at 60 °C for 10 h to constant weight.

The dried gels were ground to a coarse powder in a porcelain mortar and then transferred to a ceramic crucible. The dried gels were calcined at 550 °C for 4 h to completely remove ammonium nitrates, amines and other organic compounds. A mortar was used to finely grind the resulting charge.

Gel precursors obtained from the primary heat treatment stage were placed in Teflon autoclaves and filled with distilled water (1:35 ratio). Autoclave filling levels have been adjusted to maintain the required pressure in the system (approximately 84% of volume filled). Trace amounts of acid (0.1 N HNO_3_) were added after dispersing the system to set the desired pH. The autoclaves were placed in a thermo oven for 3–10 days at temperature of 240 °C.

The autoclaves were opened after cooling and the contents were transferred to centrifuge tubes. The liquid phase was separated by centrifugation (5 min, 15,300 rpm) and the supernatant was removed. The resulting precipitates were washed once with distilled water, separated by centrifugation and dried at 105 °C for 8 h.

### 2.2. Research Methods

X-ray diffraction (XRD) patterns were obtained using an Ultima-IV X-ray diffractometer (Rigaku, Tokyo, Japan), purchased at the expense of the Moscow State University Development Program, with D/Tex-Ultra detector, Cu–Kα radiation, scanning range of 3–65° 2θ and scanning step of 0.02° 2θ. Qualitative diagnostics were carried out using Jade 6.5 software from MDI.

FTIR spectra were obtained using a Spectrum One FT-IR spectrometer (Perkin Elmer Inc., Waltham, MA, USA) with an InGaAs detector in the middle region (4000–400 cm^−1^). To obtain a KBr pellet, 0.5 mg of the sample was dispersed in 200 mg of KBr and the resulting mixture was pressed for 20–25 min. The results were processed using the OPUS 7.0 program (Bruker Ltd., Billerica, MA, USA), and baseline correction was performed interactively by a linear method with one iteration.

The morphology features were investigated using scanning electron microscopy (SEM). To remove the accumulated charge, the sample was subjected to gold sputtering in a vacuum chamber before imaging. The studies were carried out using a scanning electron microscope LEO1450VP (Carl Zeiss, Oberkochen, Germany) in secondary electrons.

The JEOL JEM-2100F transmission electron microscope (TEM, in Tokyo, Japan) at the Tomsk Polytechnic University was employed to observe structure of synthesized kaolinites.

To evaluate the defects in the crystal structures of the synthesized kaolinites due to different synthesis conditions (charge, temperature and pH), the AGFI index was used. AGF kaolinite crystallinity indices were calculated for samples according to the following formula [19]:

(1) $$\mathrm{AGFI}=(1\bar{1}0+11\bar{1}\;\mathrm{peak}\;\mathrm{heights})/2\;\times \;(020\;\mathrm{peak}\;\mathrm{height})$$

According to the AGFI authors, ‘crystallinity’ describes the number of defects both within a layer and in their mutual arrangement (layer shifts and random translational shifts). It represents the proportions of low-defect kaolinite and high-defect kaolinite, as does the Hinckley Index [20].

## 3. Results and Discussion

### 3.1. Influence of Precursors on Synthesized Analogous Features of Kaolinite Group Minerals

The patterns of crystalline phases corresponding to natural kaolinite were obtained as a result of the XRD analysis of the samples synthesized using various Si precursors. According to the XRD data, there are no impurities, and the region of the amorphous phase reflection is covered by kaolinite reflections.

**Figure 4:**
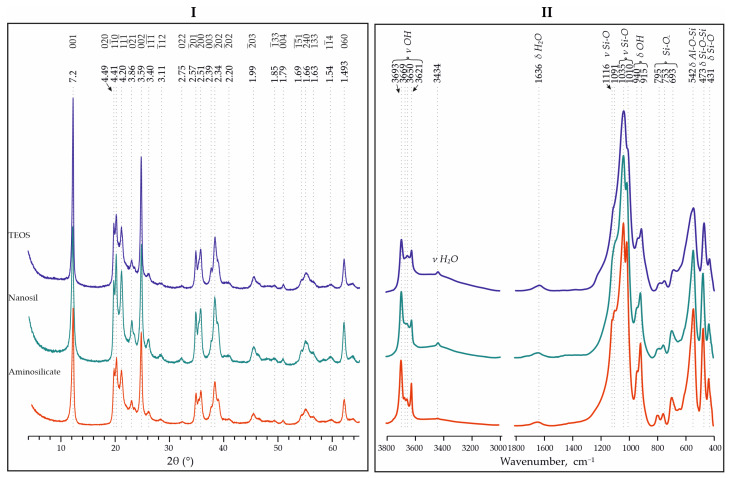
XRD (**I**) and IR spectra (**II**) for synthesized analogues of kaolinite using different precursors —TEOS, Nanosil and aminosilicate (synthesis time of 5 days and temperature of 240 °C).

Characteristic reflections of kaolinite have the following interplanar spacings: using the TEOS precursor, d = 7.21, 4.48, 3.59 and 2.38 Å; using Nanosil, d = 7.23, 4.49, 3.58 and 2.39, 2.346 Å, and using aminosilicate, d = 7.20, 4.48, 3.58, 2.39 and 2.34 Å (Figure 4).

The profile and position of the bands are characteristic of kaolinite. In the region of 3800–3000 cm^−1^, there are stretching (ν) vibrations from OH groups. The characteristic bands are 3693, 3668, 3651 and 3621 cm^−1^. The most intense bands are located in the region of 1200–400 cm^−1^: 1034 and 1010 (ν Si-O of kaolinite); 940 and 915 (deformation (δ) of OH-groups); 797, 753 and 693 (Si O_perp._); 542 (δ Al-O-Si); 472 (δ Si-O-Si) and 431 (δ Si-O) cm^−1^ [21].

The bands at 1636 and 3430 cm^−1^ correspond to the stretching and deformation vibrations of the water OH groups.

The results of the IR spectroscopy analysis revealed the presence of an amorphous phase, diagnosed by the band in the region of 1000–1100 cm^−1^. This is most likely allophane [22].

**Figure 5:**
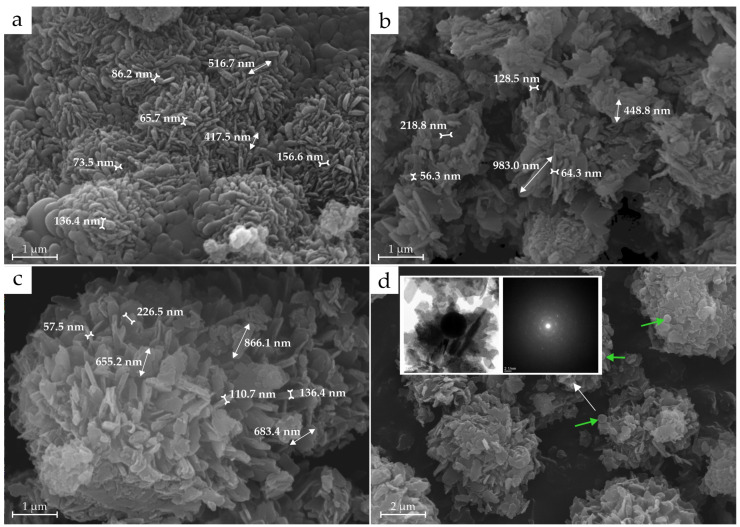
SEM micrographs for synthesized analogues of kaolinite group minerals using (**a**) TEOS, (**b**) Nanosil and (**c**) aminosilicate. (**d**) SEM and TEM micrographs of kaolinite-like spherical particles in aminosilicate are indicated by green arrows. The white arrow indicates from which particles the TEM was made.

The samples obtained with TEOS are represented by both lamellar particles, which are strongly aggregated with indistinct edges, and spherical kaolinite aggregates containing amorphous matter, as previously described by Huertas F.J. [23]. The lamellar particles are about 0.5 µm in length and are up to 0.2 µm in thickness (Figure 5a).

The synthesized kaolinites from Nanosil and aminosilicate are similar in morphology; the particles are represented by plates with clearly defined edges (Figure 5b,c). In comparison to the TEOS sample, these particles are less densely arranged, exhibiting a greater size, with lengths ranging from 0.5 to 1 µm. In the aminosilicate sample, there are rounded particles with sizes of approximately 0.2 µm (<1% in the sample), with individual spots when examined by TEM and without an amorphous halo (Figure 5d). Spherical aggregates covered with amorphous material, as seen in the TEOS sample, were not observed.

### 3.2. The Influence of Duration

The XRD patterns show a better resolution for the reflections in the region of 18–25° 2θ, with an increase in the synthesis time from 3 to 10 days for samples of the kaolinite analogues synthesized from Nanosil and aminosilicate precursors. This indicates an increase in the degree of ordering of the samples (Table 2). According to [19], all samples from Nanosil are low-defect kaolinites, while others are medium-defect kaolinites.

**Figure 6:**
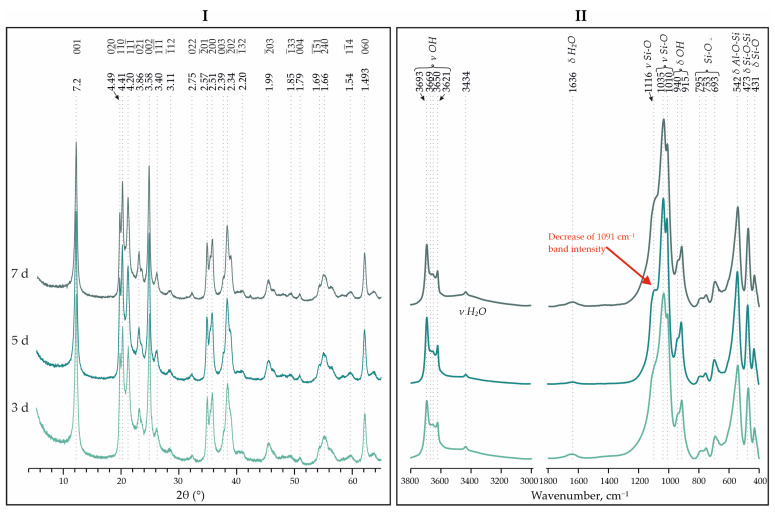
XRD (**I**) and IR spectra (**II**) for synthesized analogues of kaolinite using Nanosil with different synthesis times—3, 5 and 7 days, and temperature of 240 °C.

**Figure 7:**
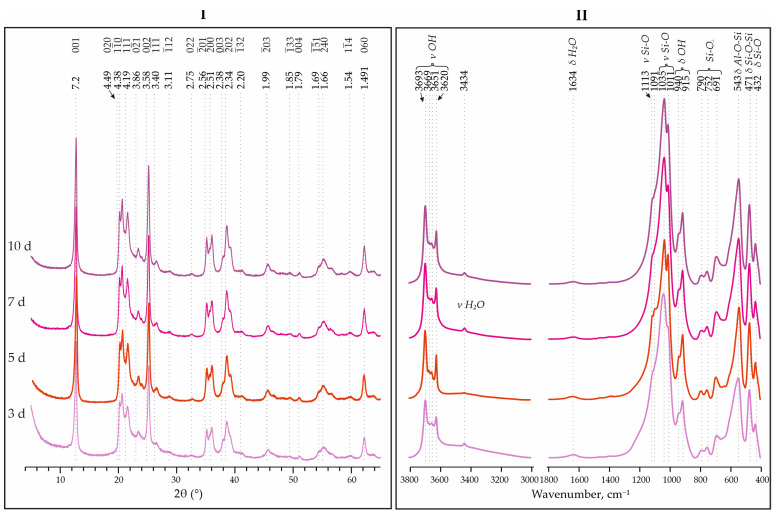
XRD patterns (**I**) and IR spectra (**II**) for synthesized analogues of kaolinite using aminosilicate with different synthesis times—3, 5, 7 and 10 days, and temperature of 240 °C.

It is also noteworthy that, in the IR spectra of Nanosil and AS, there is a decrease in the intensity of the amorphous Si-O band at 1091 cm^−1^. This indicates a better crystallization of the initial material, but the most significant changes are noticeable in the transition from 3 to 5 days of synthesis. Further increases in the synthesis time (up to 10 days) have little effect on the increases in the crystallinity index and the crystallization of the charge-starting material (Figure 6 and Figure 7).

### 3.3. The Influence of pH Conditions

**Figure 8:**
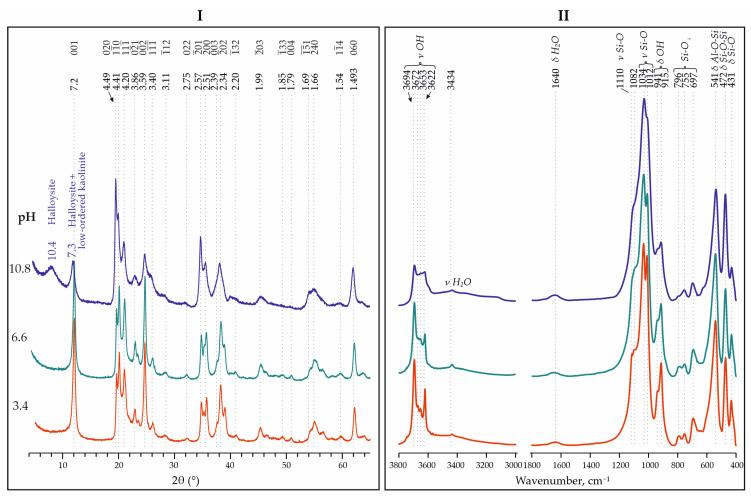
XRD patterns (**I**) and IR spectra (**II**) for synthesized analogues of kaolinite with different pH conditions—3.4, 6.6 and 10.8 (precursor—Nanosil, temperature—240 °C).

The most substantial changes are observed under the experimental conditions at different pH values. At a low pH (3.4), well-ordered kaolinite is formed, while at high pH (10.8), halloysite with a small kaolinite admixture is formed. As noted by XRD and IR spectroscopy (Figure 8), the following results were determined:

- There is a reflection with d = 10.4 Å;
- There is a shift in the reflection (001) towards a larger interplanar space—7.30 Å;
- The intensity of the reflection (020) is significantly higher than the intensity of the reflection (001);
- All the reflections are broadened and have low resolutions;
- The reflection ($\bar{1}\bar{5}1$) is well-expressed;
- The IR spectra contains bands at 3694 and 3622 cm^−1^, while the bands at 3672 and 3653 cm^−1^ are practically absent.

**Figure 9:**
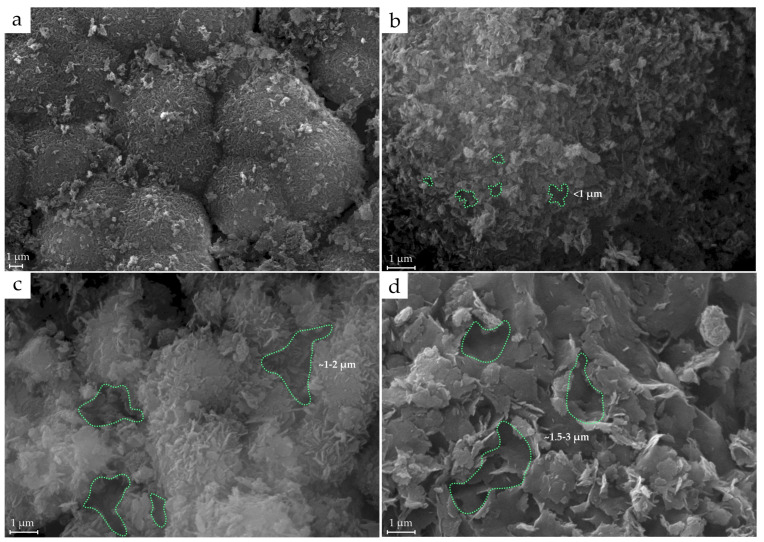
SEM micrographs for synthesized analogues of kaolinite group minerals using Nanosil precursor at different pHs: (**a**,**b**) 3.4, (**c**) 6.6 and (**d**) 10.8. Green lines indicate some of the intermicroaggregate pores.

According to the scanning electron microscopy investigations, a decrease in the crystallite sizes of the synthesized analogues of kaolinite group minerals was observed in the following range of pH synthesis conditions: 10.8 > 6.6 > 3.4 (Figure 9).

The micrographs also showed amorphous particles with signs of the beginning of crystallization. The particles are represented as rounded aggregates with diameters of 0.5–1 μm (Figure 9a). At pH = 3.4, kaolinite particles have a hexagonal shape and partial size heterogeneity (Figure 9b), and their crystallite size is ~0.5–0.8 µm. With an increase in pH of up to 6.6, the synthesized analogue of kaolinite is characterized by an increase in particle size. The crystals’ shapes also remain in the form of hexagonal platelets with distinct edges, forming large aggregates (Figure 9c). With further increases in pH of up to 10.8, the samples are mostly composed of large aggregates with a slightly curved shape, which is not characteristic of kaolinite, and most likely represents halloysite and low-ordered kaolinite. (Figure 9d). The aggregates consist of small particles with no differentiable shapes.

As illustrated in Figure 9, an increase in the pH results in a decrease in the aggregation of kaolinite particles during synthesis. The predominant type of contact also changes from contacts between kaolinite particles through their ends at a pH of 3.4 to contacts through basal surfaces. As a result, more highly porous aggregates (with smaller pores) are formed during synthesis at a low pH.

## 4. Conclusions

The formation of synthetic analogues of kaolinite group minerals with different crystallinities can be observed with the use of different silica precursors. The highest degree of crystallinity was found in the samples synthesized using the silica precursor Nanosil (AGFI—1.42). This is associated with greater homogenization at the stage of alumosilicate gel formation. In the case of TEOS (AGFI—1.16), the production of sufficiently homogeneous aluminosilicate gels for subsequent synthesis is complicated by a significant difference in the stability and chemical activity of aluminum and silica precursors in the aqueous medium at different pH values.

Apparently, in the case of aminosilicate (AGFI—1.20), when used as a precursor for silica during the first synthesis stage, a suboptimal homogenization technology was used. Despite the fact that the samples obtained have advantages in terms of the degree of crystallinity in comparison with the samples based on TEOS, it is necessary to continue to work on improving this technology.

The increase in synthesis time affected the increase in the degree of crystallinity of the synthesized analogues of kaolinite group minerals as shown by X-ray diffraction data. The infrared spectroscopy data also showed a decrease in the amount of the amorphous phase, which is an indication of greater crystallization of the substance.

The most significant changes in the synthesis samples were observed at different pH values. Well-ordered kaolinite is formed at low pH values (3.4), whereas at higher pH values—10.8—halloysite is formed with a small admixture of kaolinite, as determined by X-ray diffraction and infrared spectroscopy. According to the investigations using scanning electron microscopy, a decrease in the crystallite sizes of the formed synthetic analogues of the kaolinite group minerals was observed for a series of changes in the pH synthesis conditions (10.8 > 6.6 > 3.4).

## Figures and Tables

**Table 1:** Amounts of various precursors per 100 g of 9-aqueous Al(NO_3_)_3_ (theoretical amount of kaolinite at 100 % yield—34.4 g).

Al(NO_3_)_3_·9H_2_O, g	TEOS, mL	Nanosil (Silicasol), mL	Aminosilicate Solution (AS), mL
100	59.08	31.34	118.0

**Table 2:** Crystallinity index (AGFI) for synthesized kaolinite analogues based on different silica precursors.

Synthesis Duration	Silica Precursor Type		
	TEOS	Nanosil	Aminosilicate
3	- *	1.35	1.19
5	1.16	1.42	1.20
7	- *	1.47	1.25
10	- *	- *	1.25

* There were no samples.

## Data Availability

The original contributions presented in this study are included in the article. Further inquiries can be directed to the corresponding author.
